# Conduct after Epley's maneuver in elderly with posterior canal BPPV in the posterior canal

**DOI:** 10.1590/S1808-86942010000300005

**Published:** 2015-10-20

**Authors:** Ana Paula do Rego André, Julio Cesar Moriguti, Nathali Singaretti Moreno

**Affiliations:** 1 PhD in Medical Sciences (Biomedical Investigation) – Medical School of Ribeirão Preto – University of São Paulo (USP). Speech and Hearing Therapist – University Hospital -Medical School of Ribeirão Preto – USP; 2 Associate Professor of General Medicine and Geriatrics – Medical School of Ribeirão Preto – USP; 3 Speech and Hearing Therapist. Undergraduate

**Keywords:** aged, rehabilitation, vertigo, dizziness

## Abstract

Benign Paroxysmal Positional Vertigo is the most common peripheral vestibular disorder, especially in the elderly and presents as the predominant etiology in this population of the degeneration of the utricular macula.

**Aim:** To compare the effectiveness of the approaches after Epley maneuver. Study Design: longitudinal cohort.

**Materials and Methods:** The study included 53 volunteers with Benign Paroxysmal Positional Vertigo of the posterior semicircular canal, divided into Group 1, who underwent Epley maneuver associated with the use of neck collar and post-maneuver instructions, Group 2 underwent the Epley maneuver without the use cervical collar and/or post-maneuver restrictions, and Group 3 underwent the Epley maneuver associated with the use of a mini vibrator, without the use of neck collar and/or post-maneuver restrictions.

**Results:** In the three groups, the number of Epley maneuvers ranged from one to three. We employed the Brazilian Dizziness Handicap Inventory – pre-and post-treatment and observed a statistically significant difference on most scores pre-and post-treatment for both groups.

**Conclusion:** Regardless of the post Epley maneuver treatment selected for the treatment of Benign Paroxysmal Positional Vertigo, it was effective when comparing the Brazilian Dizziness Handicap Inventory pre-and post-treatment.

## INTRODUCTION

Benign Paroxysmal Positional Vertigo (BPPV) is one of the most frequent disorders of the vestibular system^1^. BPPV was described by Bárány^2^ in 1921 and later described in further details by Dix and Hallpike in 1952, who described its main clinical characteristics and the maneuvers which causes vertigo^3^. It is characterized by brief short duration episodes, completely disappearing in less than 45 seconds, when the head is moved to certain positions^1^,^4^, ^5^.

Paparella et al.^6^ reported that the BPPV is a condition which can appear alone or associated to other diseases – which usually triggers and/or worsens the vertigo spell. BPPV is more common in the elderly^5^,^8^, although being reported at all age ranges. It may affect both genders, but it is more prevalent among women^5^,^9^,^10^. There are many possible causes for BPPV and they vary according to author. Pereira and Scaff^11^ mention sedentary lifestyle, nutritional errors, iatrogenic (after general and otological surgery) and also report that 50% of the cases have an idiopathic origin. According to Ganança et al.^12^, BPPV can be triggered by head injury, ovarian hormonal dysfunction, metabolic and/or cardiovascular disorders, infections, after general or otological surgery, ototoxicity, syphilis, otitis media, vertebrobasilar insufficiency, psychological disorders and aging. It may also stem from other labyrinthine diseases such as, for instance, Ménière's disease, vestibular neuritis, metabolic or vascular labyrinthine disorders. Hain^13^ reports that the most common BPPV etiology in the elderly is vestibular system degeneration.

According with Caovilla et al.^14^, the pathophysiological theory for the origin of BPPV is that calcium carbonate crystals (otolith) shift position from the utriculus to the posterior, superior or lateral semicircular canals (canalolithiasis) or stick to the dome of these canals (cupulolithiasis). This abnormal shifting of otolith can be caused by any triggering factor.

For the BPPV diagnosis there are vertigo-causing maneuvers and clinical history. Two different maneuvers can be used in order to cause vertigo and nystagmus in the diagnosis of posterior canal BPPV, the Dix-Hallpike test and the Brandt-Daroff maneuver, since to observe the nystagmus duration and direction is essential for treatment^5^. Many are the approaches to be undertaken after the repositioning maneuvers proposed by Epley in 1992, which were developed along the years^15^. In his first description of the posterior semicircular canal repositioning maneuver, Epley^16^ recommends, besides the use of a bone vibrator, restrictions in terms of posture and head movements after treatment.

The mastoid vibration during the Epley maneuver, although very much advocated by some authors, is also contraindicated by others in cases of perilymphatic fistulas and in the cases of retinal shifting^17^,^18^.

The repositioning maneuver is efficient for many patients with BPPV and it is recommended as first choice approach to treatment1^9^,^20^.

Many dizzy patients deliberately restrict their activities with the aim of reducing their likelihood of developing these unpleasant symptoms, and also in order to avoid social embarrassment and the stigma they may bring about^21^.

In order to have a tool capable of establishing the profile of a dizzy patient in relation to quality of life (QL), many were the researchers who developed questionnaires and tested them in order to setup QL quantification criteria. The World health Organization (WHO) has a group of people managing it, the World Health Organization-Quality of life group^22^. Jacobson and Newman^23^ developed and validated the Dizziness Handicap Inventory (DHI), which was translated into Portuguese by Taguchi^24^ and validated by Castro^25^ (Brazilian DHI).

The present study aimed at analyzing different approaches after Epley's maneuver used to treat posterior canal BPPV in elderly patients and check the scores obtained in the Brazilian DHI before and after vestibular rehabilitation (VR), compare these scores among the groups studied.

## MATERIALS AND METHODS

The plan for the present study was submitted to the Ethical and Regulatory Standards Committee of this institution, which examined and approved it under process number 7680. 2004.

Study design: longitudinal contemporary cohort.

In the present study we had 53 volunteers, with ages equal to or higher than 60 years, from both genders. All patients who were part of this study had been diagnosed with posterior canal BPPV caused by canalolithiasis by an ENT physician by means of the Dix-Hallpike test.

Exclusion criteria: Use of medication to inhibit or stimulate vestibular function; those with other vestibular diseases – not posterior canal BPPV; age below 60 years; past of severe neck disorder which prevents the repositioning maneuver; history of retina shifting, perilymphatic fistula and BPPV by cupulolithiasis.

All the patients went through the same protocol sequence for data collection: previous consultation with an ENT; medical diagnosis of posterior canal BPPV; referral to VR; free and informed consent form; audiological anamnesis; instruction on inner ear anatomy and physiology in order for the patient to understand BPPV, vertigo assessment by means of the repositioning maneuver for the posterior canal done by a hearing therapist, according to the description of each study group; Dix-Hallpike test in order to follow treatment evolution and the time for patient discharge was established by this test when it was negative and the patient became asymptomatic; vertigo assessment by means of a post-treatment questionnaire (Brazilian DHI). There was no established time to follow occasional recurrences.

Later on, questions were asked about physical, emotional and functional aspects associated to the clinical manifestations of BPPV, and for that we used the Brazilian DHI, in which the patient answered the questions with one of the following answers: always (4), sometimes (2) and never (0) in order to check whether or not there were improvements regarding the aspects studied through the questions about symptoms after treatment. At the end, the difference between pre and post treatment scores had to be of at least 18 points, for a change to be considered significant in the self-perception of the loss caused by dizziness^25^,^26^.

The patients were selected following preset exclusion criteria and they were randomly distributed in three groups, according to an approach to be undertaken after the maneuver:

Group 1: 23 patients submitted to Epley's maneuver, followed by post-maneuver instructions (wear a neck collar for a period of 48 hours after the maneuver and follow postural restrictions).

Group 2: 15 patients submitted to Epley's maneuver, without using the neck brace and/or post-maneuver restrictions.

Group 3: 15 patients submitted to Epley's maneuver, together with the use of a North Coast Medical NC70209 minivibrator placed on the mastoid of the side affected by BPPV during the maneuver. The vibration was deployed in the frequency of 92 cycles per second, according to the international literature^27^, ^28^, ^29^. Here we did not use post-maneuver restriction instructions, nor the neck brace.

In those cases in which the Dix-Hallpike test was negative and the patient was asymptomatic, such patient was discharged from treatment. In the positive cases a new Epley maneuver was done, followed by the approach adopted in the previous treatment, until it became negative, associated to any vertigo complaint by the patient.

In order to assess the dependant variables (scores), we used a mixed effects model. The linear models of mixed effect (random and fixed effects) are used in the data analysis in which the responses from the individual are grouped and the assumption of independence between observations in the same group is not adequate^30^. These models have the assumption that their residues have a normal distribution with mean value of 0 and a variance of σ2. The proposed model is given by: yijk = η ßj αk θjk wi εijk where yijk is the observation of the dependant variable of the i-individual in the j-group and in the k-time; η is a constant (one intercepum); wi represents a random effect which captures a possible correlation between the values for the same given individual; αj is the effect of the j group (j = 1,2,3); αk is the effect of the k-time (k = 1,2); θjk is the interaction effect between the j-group and the k-time and εijk is the random error associated with the model, with N (0, σ 2) being the distribution.

The mixed effects model used in the data analysis is similar to the variance analysis (ANOVA) in two pathways; however with the difference of treating the sampled patients as a random sample coming from one population and not as fixed effects. These procedures were done by the SAS 9.0 software, the PROC MIXED. In order to study the difference between groups considering the number of variable maneuvers, we used the Kruskal-Wallis non-parametric test. The Kruskal-Wallis test is a very useful test to decide whether or not the z independent samples came from different populations, in other words, this technique proves the null hypothesis in which z samples come from the same population or from identical populations as far as the average is concerned^31^. This test is based on sample points. This procedure was carried out by the R software, using the kruskal.test command.

## RESULTS

The age of the studied sample varied between 60 and 91 years, with a mean value of 67.19 years. As far as gender goes, in the total sample of the patients, 38 (71.7 %) were females.

Of the 53 volunteers studied, 32 (60%), had left side BPPV, and 21 (40 %) had it on the right side.

Concerning our volunteers submitted to treatment, the number of maneuvers varied between one and three, with a mean of 1.3 for Group 1; 1.4 for Group 2 and 1.5 for Group 3. The maneuvers were carried out in different sessions (Table 1).

**Table 1:** Description of the number of maneuvers broken down by group

Group	Mean	Standard Deviation	Variance	Minimum	Median	Maximum
1	1,35	0,65	0,42	1	1	3
2	1,47	0,64	0,41	1	1	3
3	1,53	0,52	0,27	1	2	2

As we compare the data on the number of maneuvers, our p = 0.39; in other words, this sample provided evidence that the quantity of maneuvers is not different among the groups.

The description on the 53 volunteers as to the scores obtained in the Brazilian DHI before and after VR regarding physical, emotional, functional and general aspects shows improvements in all aspects which were studied.

By means of the modeling statistical approach, we used the mixed model (see Materials and Methods) in order to study the mean difference of each variable studied in relation to the groups (1, 2, 3 and the pre and post VR time). We notice that the physical score mean value obtained during pre-VR is different from the one obtained after VR for all the groups (p<0.0001). It is possible to notice that there was no evidence of mean physical score difference between the groups for each time, based on a significance level of 0.05 (p>0.05), with the exception of the mean difference between groups 1 and 2 in the post VR time (p=0.009) (Table 2).

**Table 2:** Difference between the groups regarding the physical subscale of the DHI

Time	Group	Mean	Group	Mean	Difference between the mean values
Pre	1	20,96	2	20,40	p= 0,764
	1	20,96	3	18,27	p=0,150
	2	20,40	3	18,27	p=0,297
Post	1	1,39	2	6,40	p=0,009
	1	1,39	3	3,60	p=0,236
	2	6,40	3	3,60	p=0,173

We notice that the mean value of the emotional, functional and general scores, obtained before VR is different from those obtained after it for all the groups (p<0.0001). It is possible to notice that there was no evidence of emotional (Table 3), functional (Table 4) and general (Table 5) mean score difference among the groups for each time studied.

**Table 3:** Difference between the groups regarding the emotional subscale of the DHI score

Time	Group	Mean	Group	Mean	Difference between mean values
Pre	1	19,04	2	17,20	p=0,533
	1	19,04	3	18,40	p=0,827
	2	17,20	3	18,40	p=0,712
Post	1	5,48	2	6,93	p=0,622
	1	5,48	3	9,87	p=0,141
	2	6,93	3	9,87	p=0,368

**Table 4:** Difference between the groups regarding the functional subscale DHI score

Time	Group	Mean	Group	Mean	Difference between mean values
	1	22,09	2	21,60	p=0,855
Pre	1	22,09	3	22,53	p=0,867
	2	21,60	3	22,53	p=0,750
	1	5,48	2	9,47	p=0,138
Post	1	5,48	3	8,93	p=0,198
	2	9,47	3	8,93	p=0,856

**Table 5:** Difference between the groups regarding the general subscale DHI score

Time	Group	Mean	Group	Mean	Difference between mean values
Pre	1	61,91	2	59,20	p=0,675
	1	61,91	3	59,20	p=0,675
	2	59,20	3	59,20	p=1,000
Post	1	12,35	2	22,80	p=0,110
	1	12,35	3	22,40	p=0,124
	2	22,80	3	22,40	p=0,955

## DISCUSSION

The age of the population studied varied between 60 and 91 years, with a mean value of 67.2 years. These data are similar to those obtained from the population studied by Ganança et al.^21^, who found a mean age of 66.9 years. The very aging-related alterations in the body balance system, the greater prevalence of chronic-degenerative disorders and the chronic use of medication foster the development of dizziness in this population^21^.

In relation to the gender of the volunteers studied, there was a prevalence of females, with 38 volunteers (71.7%) of the total sample, which is in agreement with other reports in the literature^9^,^11^,^32^, ^33^, ^34^ which reported a predominance of body balance disorders and BPPV in females. According with Guzmán et al.^35^ such findings are associated to hormonal alterations frequently found among women.

In this study there was a prevalence of unilateral involvement (100%) in our volunteers, matching results reported by Ganança et al.^12^ – 88.2% of unilateral involvement.

Concerning the side of the involved canals, we found 32 (60%) left-side posterior canals and 21 (40%) volunteers with right-side involvement. This finding can be explained by the fact that the BPPV diagnosis was done by different ENT physicians of this clinic, and we did not control the side used for the assessment; thus, disagreeing from the reports of some authors ^33^,^36^, ^37^, ^38^ who report a prevalence on the right side, explained by the fact that the first maneuver is usually performed on the right side whenever the patient did not know which side triggered vertigo or nystagmus, as recommended by Slater^39^. It is also contrary to findings from Dorigueto et al.^40^ who reported that both sides are equally affected. It matched findings from Mantello et al.^41^ and an explanation for such finding was not found in the literature studied.

Of the 53 volunteers treated, 100% were discharged from VR; nonetheless, the number of necessary interventions for such treatment varied between one and three.

As to the number of Epley's maneuvers used for the treatment of the volunteers, in Group 1 the mean value as 1.3; in Group 2 we needed 1.4 in average and for Group 3, 1.5 maneuvers were necessary for complete symptom remission, seen upon a negative Dix-Hallpike test. These findings match those from Barreto^42^, who reports that symptom remission and a negative Dix-Hallpike is achieved, according to the literature, in 44 to 89% of the cases with one single intervention. This number goes up to 100% with up to 4 interventions^42^.

It is known that recurrences may happen after months or years of cure; nonetheless, in our study we did not analyze symptom recurrence. Notwithstanding, it is necessary to carry out a longitudinal study with these patients after treatment discharge in order to characterize whether or not the post-Epley maneuver differences used for the treatment can impact on the recurrence.

The physical aspects of the Brazilian DHI assesses the relationship between the onset and/or worsening of dizziness with eye movements or head and body movements; and these are common symptoms in cases of BPPV, which justifies the greater involvement of the physical score in this study^21^. Enloe and Shields^43^ and Roberson and Irland^44^, observed that the physical and functional aspects were more compromised than the emotional aspect, and our findings match those from these authors. Regarding the difference seen between groups 1 and 2 after treatment for the physical variable, we did not find in the literature studied any mention which would justify such finding.

In analyzing the quality of life using the questionnaire (Brazilian DHI), we can consider that the VR provided an increment in the quality of life both for the physical aspect as well as functional and emotional, concerning the PBBV volunteers assessed in this study.

The treatment of BPPV with VR provided a statistically significant improvement in the QL of the volunteers in this study, and this result was established by the statistical analysis of the paired data of the handicap questionnaire for dizziness in the general aspect as well as physical, emotional and functional aspects.

Our results show that there is no difference in VR efficacy between the post Epley maneuver approaches used, which allows us to assume that the treatment which is truly effective to treat BPPV of the posterior canal caused by canalolithiasis in the elderly is the Epley maneuver alone, without associated restrictions. These findings match those reported by Simoceli et al.^45^ who discuss the efficacy of the Epley maneuver used without restriction instructions and neck brace are to avoid the incorrect shifting of the otoliths or its debris after the maneuver, since the period without head movement would facilitate the absorption or adherence of the otoliths to the utriculus. Nonetheless, such restrictions can cause discomfort to the patient preventing him/her to perform daily activities38, there are also reports of people who had torticollis after wearing the neck brace^45^.

According with Zucca et al.^46^, head movement restrictions would not be very relevant in the first 24 hours after employing the Epley Maneuver, since under normal conditions of calcium volume in the endolymph, the otoliths dissolve along five to twenty hours. This anatomical and physiological aspect could explain why it is not necessary to wear the neck brace and/or cause restrictions to head movements.

## CONCLUSION

Based on the critical analysis of the results obtained and the analysis of the objectives proposed one can conclude from this study that regardless of post-maneuver approaches used to treat posterior canal BPPV in elderly patients, by means of using VR, were efficient to treat the elderly voluntary considering the results from the negative Dix-Hallpike test at the end of the treatment. We have also noticed that the truly efficient treatment of posterior canal BPPV in the elderly is the maneuver alone, without the need for movement restrictions.

The number of necessary maneuvers, regardless of group, did not show statistically significant difference, with a trend towards a larger number in the group which used the vibrator. Nonetheless, we believe there is a need to run a longitudinal trial with these patients who did not wear the neck brace and were not submitted to movement restriction or instructions, so as to be able to study possible recurrences.
